# Is smart carbon emission reduction justified in China? Evidence from national big data comprehensive pilot zones

**DOI:** 10.1016/j.heliyon.2023.e17744

**Published:** 2023-06-28

**Authors:** Han Bu, Guomin Li, Xiangyu Yu, Zhou Xun

**Affiliations:** a School of Economics and Management, Southwest University, China; b School of Economics, Nanjing University of Finance and Economics, China; c School of Economics, Jiangxi University of Finance and Economics, China; d UniDT Technology (Shanghai) Co., Ltd, China

**Keywords:** New digital technologies, Carbon emissions, Industrial structure, Green technology innovation, Industrial agglomeration

## Abstract

The intelligent revolution caused by new digital technologies has provided new impetus to reduce carbon emissions. However, the current research on new digital technologies and carbon emissions is still in its infancy and lacks empirical conclusions between them. Therefore, this paper studies the impact of new digital technologies on carbon emissions, identifies its mechanism, and analyzes the regional heterogeneity of its effects. This research treats the National Big Data Comprehensive Pilot Zones pilot in China as a quasi-natural experiment for the development of new digital technologies along with city-level data covering from 2011 to 2019 to conduct a staggered difference-in-difference (DID) model analysis. We find that new digital technologies significantly reduce carbon emissions. This conclusion is still valid after a series of robustness tests such as heterogeneity treatment effect analysis, ex-ante trend test, spillover effect test, and placebo test. Additionally, new digital technologies can reduce carbon emissions by promoting the transformation of industrial structure, improving the level of green technology innovation, and promoting industrial agglomeration. At the same time, the heterogeneity analysis shows that new digital technologies' carbon emission reduction effect is more evident in non-western regions, southern regions, and large cities. To expand the carbon emission reduction effect of new digital technologies, the government should promote the development and application of new digital technologies, and implement differentiated policies based on regional characteristics.

## 1 Introduction

China's high-speed economic growth over the past few decades has come at the cost of a significant increase in carbon emissions (CE). Data indicates that China's CE have reached 9.9 billion tons in 2020, accounting for 31% of global emissions - significantly higher than the United States' 13.8% [1]. This CE increase is a key contributor to global warming [2]. Global warming has brought about serious issues, including imbalanced ecosystems, frequent natural disasters, unhealthy living conditions, and increased income inequality [[3], [4], [5]], threatening the sustainability of human society. To cope with adverse climate change, at the 75th United Nations General Assembly in 2020, China promised to reach the peak of CE by 2030 and strive to achieve carbon neutrality by 2060. Achieving these “dual carbon” goals will play a crucial role in the global response to climate change. However, China faces the challenging task of achieving these goals, particularly in light of the complex international economy and the ongoing impact of COVID-19, during a critical period of economic transformation and slowing economic growth. In this context, further discussion of the factors and mechanisms affecting CE has become the crucial topic to the transformation of China's manufacturing intensive economy to a low-carbon economy.

Sustaining economic growth while reducing CE is the central consideration of economic development in China [6]. However, China's urbanization and industrialization have not yet been completed, and many low-income groups still exist. Hence, China's efforts toward low-carbon development cannot undermine its economic growth. It is imperative that China shifts its economic growth model and embraces new growth drivers, such as the digital economy (DE) development holds great potential in achieving the “dual carbon” goals while driving economic growth [[7], [8], [9]]. China's DE is booming, accounting for 38.6% of the GDP in2021.^1^ The fundamental reason why DE impacts CE is rooted in the widespread adoption of digital technologies (DT) across the economy and society. However, the existing research mainly focuses on analyzing the impact of DE on CE and the role of traditional digital technologies (TDT) such as mobile communications, computers, and the Internet in CE. The role of new digital technologies (NDT) represented by cloud computing, the Internet of Things (IoT), Big Data, machine learning, and Artificial Intelligence (AI) in CE has not yet attracted enough attention.

In fact, there is still controversy over the role of DE and TDT in reducing CE [[10], [11], [12], [13], [14]]. The reason for the debate is that the specific impact of DE on CE is the combined result of TDT and NDT, and the rapid industrialization brought by TDT may increase CE [12]. Compared with TDT, NDT have more efficient data processing and information transmission capabilities, as well as deeper data analysis and application capabilities, which can adapt to different production modes and demonstrate enormous potential in green production. These NDT display more advanced and intelligent features and are increasingly taking over tasks that were once performed by humans [[15], [16], [17], [18]]. The intelligent revolution brought about by NDT may become an important opportunity to achieve CE targets in the future. However, the environmental effects brought by NDT have yet to be systematically discussed.

This paper aims to comprehensively explore the role of NDT in CE from the perspective of Big Data. First, other new digital technologies rely on Big Data as a basis for processing, analysis, and decision-making. The interdependence between these technologies and Big Data leads to the enhancement of value creation and technology capabilities. For example, Big Data enhances the ability of the IoT to handle complex data [19], and supports the development of machine learning and AI [20,21]. Meanwhile, the powerful computing and storage capabilities of cloud computing, combined with the data analysis abilities of machine learning and AI, contribute to the further development of Big Data [[20], [21], [22]]. Second, implementing China's National Big Data Comprehensive Pilot Zones (NBDCPZ) pilot policy provides a favorable opportunity to study the relationship between Big Data development and CE. Implementing the NBDCPZ pilot policy is relatively exogenous to urban CE. The original intention of the construction of the NBDCPZ is to explore the management and sharing of data resources and the application of digital resources to play its radiating and demonstration role, rather than reduce CE.

This study employs a panel data set comprised of 282 cities in China at the prefecture level and above from 2011 to 2019 to examine the effect of NBDCPZ pilot policy on CE through a staggered difference-in-difference (DID) method. Compared with previous studies, this paper has three main contributions. First, it broadens the scope of research regarding factors that impact carbon emissions. While prior studies have produced substantial results in the examination of causes of CE and the role of ICT, digital finance, and DE in such emissions, the impact of NDT on CE remains under-investigated. Second, this paper takes the fundamental fact that Big Data can significantly promote the development of NDT as its basis, and explores the environmental impact of NDT from the perspective of Big Data, thereby enriching the research perspective of NDT. Additionally, a unique quasi-natural experiment-the NBDCPZ pilot project in China is employed to aid in the accurate evaluation of the causal relationship between the development of NDT and their economic and social impacts, providing valuable reference points for subsequent research. Lastly, this study outlines a comprehensive theoretical framework to analyze the direct and indirect mechanisms of the impact of NGT on CE, which is then empirically tested. The results of this investigation provide a valuable reference for policy-makers in effectively reducing CE and furthering the development of NDT.

The rest of this paper is arranged as follows: Section 2 provides a literature review and the policy background; Section 3 fully expounds the theoretical analysis and puts forward the research hypotheses; Section 4 presents research methods and data; Section 5 discusses the empirical results and robustness tests; Section 6 verifies the mechanism; Section 7 presents the heterogeneity analysis and the last section draws conclusions and policy recommendations.

## 2 Literature review and policy background

### 2.1 Literature review

In recent years, research topics concerning TDT and its social-economic impacts have attracted great interest and attention of scholars. However, a consensus has yet to be reached. For example, Ozcan and Apergis (2018) explored the role of ICT on CE in 20 new economies [23]. This research proves that the Internet significantly reduces CE. Lu (2018) and Haini (2021) reached the same conclusion using data from Asian countries and ASEAN countries respectively [24,25]. In addition, Liu et al. (2022) also found that DT has a significant positive externality in CE [26]. However, DT itself will consume much energy [27]. DT will further promote industrialization, thus increasing energy consumption and generating much electronic waste [12]. Therefore, some studies believe that DT will not significantly impact CE and may even negatively impact the environment by releasing more CE. Zhang and Liu (2015) thought ICT can not impact CE in the China's west regions [28]. Asongu et al. (2018) used the data from 44 countries in sub-Sahara Africa (SSA) from 2000 to 2012 and the generalized moment estimation (GMM) model to find that the use of ICT is insignificant on reducing CE [10]. In addition, Lee and Brahmasrene (2014) and Salahuddin et al. (2016) analyzed the impact of DT on CE using panel data from 1990 to 2019 of nine member countries of the ASAN and 1991 to 2012 of OECD countries, respectively [29,30]. The research results show that Internet use has significantly increased CE. Raheem et al. (2020) used the PMG method to explore the impact of ICT on CE. This study further found that ICT has a long-term positive effect on CE in G7 countries [12].

In addition, the potential of NDT in reducing CE has received much research attention. However, these studies only focus on its technical possibilities. For example, Maksimovic (2018) argues that deploying green signals and system models in the Internet of Things systems can reduce CE during information transmission [31]. Lamba and Singh (2019) used Big Data technology to develop an operator selection model that includes carbon dioxide generation during ordering, holding inventory, production, loading, unloading, and transportation, thereby reducing carbon emissions [32]. Aldossary and Alharbi (2021) found that optimizing the location of requested resources and applications can reduce CE from cloud computing [33]. Zhang et al. (2022) found that applying Big Data technology can help enterprises accurately find suitable emission reduction partners to promote enterprises' CE reduction cooperation effectively [34]. Meanwhile, a small amount of research has discovered evidence from the perspective of AI that NDT can improve CE efficiency and reduce CE intensity [35,36].

To sum up, most studies have explored the relationship between TDT and CE from various perspectives based on different backgrounds. In contrast, few studies have systematically explored new digital technologies' carbon emission reduction effects. In addition, although a few studies have recognized the possible impact of NDT on CE, they lack sufficient theoretical analysis and practical empirical tests. How NDT affect carbon emissions needs to be further clarified. At the same time, the existing research failed to find an appropriate way to measure the development of NDT which also led to the insufficient empirical evidence to understand the impact of NDT on CE.

### 2.2 Policy background

In the agricultural era, land and labor were essential production factors. After the industrial revolution, capital became the most crucial factor of production. With the rapid development of NDT, data has not only become a new production factor, but also an essential resource and strategic resource. Access to high-quality data is the core demand for developing new digital technology. Major industrialized countries have successfully launched Big Data development strategies to further promote the NDT development.

To promote the NDT development and improve international competitiveness, China has always been committed to promoting the Big Data development. In general, China's Big Data development went through three stages. The first stage is the preliminary stage initiated before 2014. It was more about the discussion of big data concepts and technologies, and failed to reach a complete consensus. The second stage is the landing stage. This stage is between 2014 and 2019. The development agenda of Big Data has risen to the national strategy. The NBDCPZ has been promoted in an orderly manner, and policies related to big data have been introduced in various provinces. The third stage is the deepening stage, which is post 2019. Data has officially become a new production factor, and it is clearly pointed out that the data factor is the key factor for DED.

Among them, constructing NBDCPZ is a meaningful step for China to promote the big data development into practical application, which is of milestone significance. At present, the NBDCPZ are divided into two batches approved for construction. The first batch of construction list is Guizhou Province, which was approved to become the first NBDCPZ construction region in China in 2015. The second batch of construction lists were released in 2016, including Beijing-Tianjin-Hebei, Pearl River Delta, Shanghai, Henan, Chongqing, Shenyang and Inner Mongolia. So far, there are eight big data comprehensive pilot zones in China, which will jointly lead the Big Data development in the “four major sectors” of the east, the middle, the west, and the northeast, to achieve data sharing, coordinated development within the region, and accelerate industrial transformation. The main goal of setting up the Big Data pilot zone is to effectively break the data resource barrier, strengthen infrastructure planning, create a batch of advanced Big Data products, cultivate a batch of Big Data backbone enterprises, build a batch of ample data mass innovation space, and cultivate a batch of considerable data industry talents, to effectively promote relevant institutional innovation and technological innovation, and explore the value of data resources.

It can be seen from the development goals of the pilot zones that the implementation of NBDCPZ is not only to develop the Big Data industry itself but also to promote the NDT development further and strengthen the integration of NDT and the real economy. New digital technologies mainly depend on data elements and modern communication technology. High-quality data and excellent information infrastructure are indispensable for bringing NDT into full play. The ten major projects in the NBDCPZ mainly include “mass innovation Big Data project,” “Big Data key technology and product R&D and industrialization project,” “industrial and emerging industry Big Data project,” “information infrastructure improvement project,” etc. Therefore, establishing the NBDCPZ can deepen data mining and application and promote the NDT development such as machine learning, IoT, AI, and intelligent robots.

## 3 Theoretical analysis and research hypotheses

### 3.1 Direct impact of new digital technologies on carbon emissions

With the continuous development of NDT with data as the core, NDT will play a driving role in reducing CE. Specifically, the impact of NDT on CE is mainly reflected in three aspects: promoting the low-carbon transformation of enterprise production mode, promoting the green low-carbon transformation of lifestyle, and innovating the government governance model.

First, from the production perspective, NDT such as Big Data, machine learning, and AI can improve the efficiency of R&D investment, reduce R&D costs, and accelerate the development of green production technologies. These technologies also facilitate establishing an energy management system, optimizing production processes, and minimizing energy consumption in the production process. For instance, machine learning and artificial intelligence can simulate or collect data to adjust and optimize production parameters, thereby reducing unnecessary energy consumption.

Secondly, regarding the transformation of lifestyles, NDT such as Big Data and AI can effectively evaluate personal credit, track personal actions promptly, and accurately predict personal behaviors, thus significantly reducing the search cost, as well as search-related carbon emissions [37]. Moreover, NDT enable smart travel and intelligent homes, thereby increasing energy efficiency in transportation and households and reducing CE.

Finally, applying NDT can improve governance efficiency through the rapid collection, collation, and analysis of data, thereby reducing CE. For example, the government can use digital Big Data platforms to explore extensive enterprise information to formulate practical economic policies, reducing information search costs and promoting CE reduction [38]. Moreover, the carbon emission measurement technology development based on NDT can enhance the government's carbon emission management capability by conducting data statistics, real-time monitoring, and accurate analysis of CE.

The above analysis leads to the formulation of the following research hypothesis.

Hypothesis 1 NDT can significantly reduce CE.

### 3.2 New digital technologies, industrial structure transformation and carbon emissions

Technological progress often accompanies industrial structure transformation (IST) [39]. Therefore, applying NDT is expected to bring about changes in the industrial structure. These changes are brought about by three factors: digital industrial structure optimization and upgrading, traditional industrial structure optimization and upgrading, and changes in consumer demand for products and services.

First, NDT further optimize and upgrade the digital industrial structure. Digital industrialization can promote the development of emerging industries such as cloud computing, Big Data, IoT, and AI and promote the digital industrial structure optimization and upgrading. For example, integrating traditional digital industries, such as 5G technology, with NDT will enable more efficient monitoring and optimization of energy consumption during data transportation and analysis.

Second, NDT further optimize and upgrade the traditional industrial structure. The production process of traditional industries can be optimized and upgraded by combining with NDT, thus causing its further intelligent transformation. More than this, applying NDT can reasonably allocate production factors and improve the degree of integration between industries, thus promoting industrial transformation [40]. For example, IoT, Big Data, AI, and other NDT for data collection, analysis, and intelligent management in the energy industry can help master the power generation of wind power and photovoltaic, promote the matching of power transmission and energy storage, and reduce wind and light abandonment.

Finally, applying NDT on the consumer side will further change the demand structure of the product and service market, thus forcing industrial structure optimization and upgrading. The ability of consumers to frequently and effectively enjoy the benefits of intelligent products and services will increase the demand for such products and services. To remain competitive in the market, enterprises will have to optimize and upgrade their industrial structure to produce products and services that meet the demands of the digital economy era.

Meanwhile, IST is a critical factor in the effort to curb CE [41]. IST involves the redirection of resources such as human capital, physical capital, and energy from traditional industries towards emerging and high-tech industries. This shift towards new industries and technology contributes to the increase in green and renewable energy sources, as well as the reduction of fossil fuels. Consequently, this leads to an amelioration of the energy consumption structure and a reduction in energy consumption. In addition, In the process of IST, the enhancement of linkage between different industries and the same industry can improve the efficiency of resource factor utilization [42].

The above analysis leads to the formulation of the following research hypothesis.

Hypothesis 2 NDT can reduce CE by promoting IST.

### 3.3 New digital technologies, green technology innovation and carbon emissions

The role of digital technology in promoting green technology innovation (GTI) has been extensively studied in recent years [43,44]. The continuous advancement of digital technology has resulted in the emergence of NDT such as Big Data, intelligent robots, and AI, which have the potential to boost green technology development significantly.

First, applying NDT can optimize the human capital structure of enterprises, thus improving green technology innovation capability. Human capital is widely recognized as the core driving force for enterprise GTI [45]. The widespread use of NDT in various industries creates a demand for high-tech and highly educated talents, leading to human capital structure optimization.

Second, applying NDT has the potential to ease financing constraints, thus promoting GTI. Financing constraints and the availability of funds play a critical role in the GTI of enterprises [46]. On the one hand, using NDT can help enterprises to the transformation of traditional production to intelligent production, reducing various costs and improving resource allocation efficiency. On the other hand, it can help financial institutions to accurately analyze the business status and future development trends of enterprises, solving the problem of information asymmetry and providing credit funds for enterprise development.

Finally, integrating NDT can enhance the knowledge spillover effect, which is crucial in promoting GTI [47]. New digital technologies break down the barriers of information flow between enterprises, enabling enterprises to collect and analyze the market demand for low-carbon technologies and products in a timely and accurate manner, leading to improved green technology innovation.

Meanwhile, the adoption of green technology has been recognized as a promising solution to the challenge of environmental pollution. Research shows that GTI can significantly reduce CE [[48], [49], [50]]. On the one hand, the widespread use of green technology in both industrial production and daily life can promote cleaner production and consumption, increase energy efficiency, and reduce CE from both the production and consumption sectors, thus achieving source control of CE. On the other hand, GTI can drive the development of new energy fields, such as photovoltaics, wind power, and renewable energy, and encourage a transition to a greener, low-carbon, and clean energy consumption structure, ultimately leading to a direct reduction of CE.

The above analysis leads to the formulation of the following research hypothesis.

Hypothesis 3 NDT can reduce CE by improving GTI.

### 3.4 New digital technologies, industrial agglomeration and carbon emissions

Industrial agglomeration (IA) can effectively reduce the cost of three different types of transportation, namely, mobile goods, people, and knowledge [51,52], which makes the location selection between enterprises show the characteristics of agglomeration. The application of NDT will also affect the location of enterprises through the transformation of commodities, human capital, and knowledge.

First, NDT give rise to new products, services, and demand, allowing enterprises to congregate in the digital technology industry and reduce the cost of transportation associated with obtaining relevant products and services. For instance, the intelligent platform generated through the advancement of Big Data and AI technologies can enhance enterprise efficiency [53]. To take full advantage of these products and services, enterprises prefer to locate themselves in close proximity to providers of related products and services.

Second, developing and applying NDT fosters a talent pool with digital skills, leading to an increase in the number of traditional enterprises enrolled in the digital technology industry. The proximity of these enterprises enables the sharing of labor. The process of traditional enterprise transformation into intelligent enterprises requires a substantial amount of digital-skilled labor force, thereby attracting more traditional enterprises to converge in the digital technology industry.

Finally, NDT promote technological innovation through the acceleration of knowledge creation and technology spillover, as well as improving learning ability [54], enabling enterprises to gather in the digital technology industry to acquire NDT and enhance their innovation capabilities. The convergence of different enterprises in the digital technology industry will result in a further acceleration of innovation and improvement of product competitiveness.

Meanwhile, IA can reduce CE through technological and structural impacts [[55], [56], [57]]. IA fosters the exchange of knowledge and information among enterprises, thereby enhancing their innovation and technological capabilities. The improvement in enterprise innovation and technology leads to a reduction in total carbon consumption, increased energy utilization efficiency, and a more optimized energy consumption structure, all of which ultimately result in decreased CE.

The above analysis leads to the formulation of the following research hypothesis.

Hypothesis 4 NDT can reduce CE by promoting IA.

**Fig. 1:**
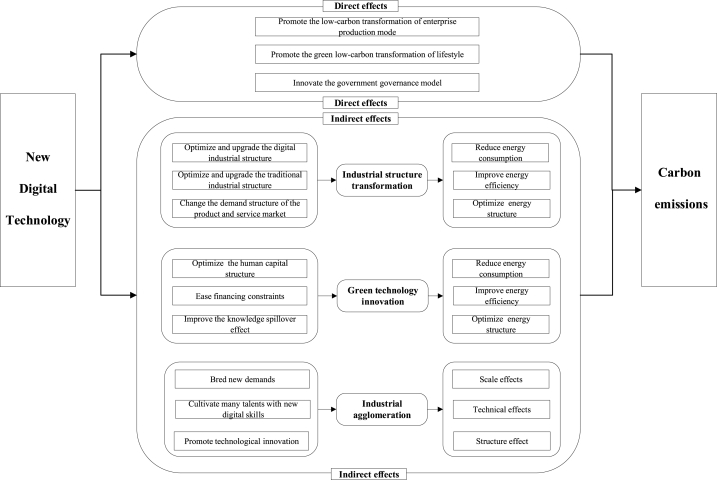
Theoretical analysis framework.

The overall theoretical analysis framework is shown in Fig. 1.

## 4 Methods and data

### 4.1 Model

#### 4.1.1 Baseline model

The implementation of NBDCPZ has comprehensively changed the level of NDT in pilot cities, which provides an excellent quasi-natural test to identify the impact of NDT on CE. Considering that the listed NBDCPZ cities are not approved simultaneously, we draw on Bertrand and Mullainathan (2003) to construct a staggered DID model to test the net effect of NDT on CE [58]:

(1) $$\mathrm{LN}\;{\left(TCE\right)}_{ct}=\partial_{0}+\partial_{1}{NBDCPZ}_{ct}+\gamma z_{ct}+\tau_{t}+\delta_{c}+\varepsilon_{ct}$$

where c and t represent city and year respectively. $\mathrm{LN}\;{\left(TCE\right)}_{ct}$ is the logarithm of total CE. ${NBDCPZ}_{ct}$ is the dummy variable. If city c is set up as NBDCPZ in the t year, ${NBDCPZ}_{ct}$ equals 1, and 0 otherwise. $z_{ct}$ is a series of control variables. $\tau_{t}$ and $\delta_{c}$ control the fixed effects of year and city respectively. $\varepsilon_{ct}$ is the error term, and the standard error is city clustering standard error. $\partial_{1}$ characterizes the net effect of the NBDCPZ on CE. If the value of $\partial_{1}$ is significantly negative, it indicates that NDT are conducive to reducing CE; otherwise, there is a promoting effect.

#### 4.1.2 Mediating effect model

In order to test the impact mechanism of NDT on CE, this paper is based on the approach of Baron and Kenny (1999) [59], taking industrial optimization and upgrading, green technology innovation, and industrial agglomeration degree as intermediary variables, and builds mediation effect models to test the impact mechanism. Firstly, repeat model (1) as the first step given in Model (2). Second, regression with the intermediary variables of industrial optimization and upgrading, green technology innovation, and industrial agglomeration degree to NBDCPZ to build the model (3). Finally, the intermediary variable is put into the model (1) to establish model (4). When $\partial_{1}$、 $\delta_{1}$ and $\beta_{1}$ are significant, if $\sigma_{1}$ is smaller or significantly lower than $\partial_{1}$, it indicates that there is a mediation effect.

(2) $$\mathrm{LN}\;{\left(TCE\right)}_{ct}=\partial_{0}+\partial_{1}{NBDCPZ}_{ct}+\gamma z_{ct}+\tau_{t}+\delta_{c}+\varepsilon_{ct}$$

(3) $${MID}_{ctk}=\delta_{0}+\delta_{1}{NBDCPZ}_{ct}+\vartheta z_{ct}+\tau_{t}^{1}+\delta_{c}^{1}+\psi_{ct}$$

(4) $$\mathrm{LN}\;{\left(TCE\right)}_{ct}=\sigma_{0}+\sigma_{1}{NBDCPZ}_{ct}+\sigma_{2}{MID}_{ctk}+\varphi z_{ct}+\tau_{t}^{2}+\delta_{c}^{2}+\upsilon_{ct}$$

### 4.2 Variable selection

#### 4.2.1 Dependent variable

**Fig. 2:**
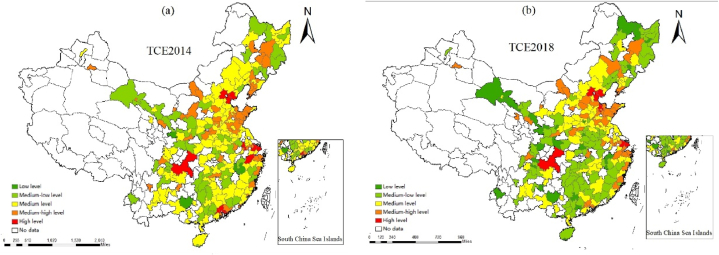
Spatial distribution of China's CE in 2014 and 2018.

The logarithm of total CE (LN(TCE)). In this paper, LN(TCE) is composed of CE generated by electricity, coal gas, liquefied petroleum gas (LPG), transportation and heat consumption. Among them, CE from electricity, coal gas, and LPG were calculated using the method proposed by Glaeser and Kah (2010) [60]; CE from urban transportation consumption were calculated using the method proposed by Li et al. (2013) [61]; and CE from thermal energy consumption were calculated using the method proposed by Wu and Guo (2016) [62]. Fig. 2 shows LN(TCE) of prefecture-level cities in China in 2014 and 2018, directly showing the differences in CE between regions. In addition, it can be intuitively found from the figure that China's CE have been reduced to a certain extent.

#### 4.2.2 Independent variable

The NBDCPZ pilot policy (NBDCPZ).^2^ If a city is listed as NBDCPZ, and the data comes from after the year of policy implementation, the value of NBDCPZ is 1. Otherwise, it is 0. In this sample, 67 cities from western, central and eastern China are located in the NBDCPZ, which means that the pilot policy is implemented universally across the country but distributed with certain degree of randomness.

#### 4.2.3 Intermediary variables

(1) Industrial structure upgrading (ISU) and optimization (ISO). Following the practice of Cheng et al. (2018) [63], we use the proportion of the added value of the tertiary industry in GDP to measure ISU and use the reciprocal of the Theil index to measure ISO.
(2) Green technology innovation (GTI). R&D expenditure, new product sales revenue, and patents are widely used to assess technological innovation [64]. However, it is difficult to obtain data on R&D expenditure on green technology innovation and sales revenue of new products. To this end, we use the number of green patent applications per 10,000 people in the city as a proxy variable for GTI.
(3) Industrial agglomeration degree (IAD). We follow the practice of Ke and Yu (2014) and measure the regional industrial agglomeration by the employment density of each region [65]. The greater the employment density of a region, the higher the industrial agglomeration of this region.

#### 4.2.4 Control variables

To minimize the deviation caused by missing variables on the benchmark results, we refer to Yu et al. (2022), and Li and Wang (2022) and set the following control variables [14,66].

(1) Economic development level (ln(GDP)): logarithm of GDP
(2) Human activity (ln(TP)): logarithm of the total population at the year's end
(3) The degree of openness (FDI): ratio of the number of foreign-invested enterprises
(4) The level of financial development (FDL): ratio of loans outstanding by financial institutions
(5) The degree of government intervention (FE): proportion of revenue in the local general budget to GDP
(6) Green area (GC): proportion of green area
(7) Environmental Pollution Index (EPI): Comprehensive index calculated by entropy weight method based on industrial waste water, exhaust gas, solid waste, sulfur dioxide and soot emissions

### 4.3 Data source

Based on the availability of data, our research sample consists of data from 282 prefecture level cities in China from 2011 to 2019. These data are mainly collected from the China Statistical Yearbook, China Urban Statistical Yearbook, China Urban Construction Statistical Yearbook, China Environmental Statistical Yearbook, EPS database, national intellectual property database, etc. In addition, the price-related variables will be converted into prices in 2011 according to the corresponding price index. Table 1 reports some basic statistical results of the main variables.

**Table 1:** Descriptive statistics.

VARIABLES	N	mean	sd	min	max
LN(TCE)	2538	6.330	1.143	2.451	9.533
NBDCPZ	2538	0.107	0.309	0	1
ln(GDP)	2538	16.575	0.925	14.106	19.760
ln(TP)	2538	5.888	0.698	2.970	8.136
FDI	2500	0.043	0.051	0	0.335
FDL	2538	0.990	0.615	0.118	9.622
FE	2538	0.079	0.028	0.023	0.227
GC	2538	0.399	0.053	0.028	0.648
EPI	2391	0.073	0.076	0.010	0.729

## 5 Results

### 5.1 Baseline results

We use model (1) to test the CE effect of NDT, and Table 2 reports these results. Column (1) controls only urban and year fixed effects; Column (2) controls the economic variables at the urban level based on the regression mentioned above; Column (3) further controls the environmental variables at the urban level. These results all indicate that establishing the NBDCPZ can significantly reduce CE, which is coherent to theoretical expectations. According to the result in column (3), compared with control cities, CE of the experimental cities have decreased by 9.8% (exp (−0.104) - 1) after establishing the NBDCPZ, which shows that NDT have indeed significant potential in reducing CE. In general, the empirical results of the stepwise regression in Table 2 support the theoretical expectations of this paper, and Hypothesis 1 is supported empirically.

**Table 2:** Baseline estimation results.

VARIABLES	(1)	(2)	(3)
	LN(TCE)	LN(TCE)	LN(TCE)
NBDCPZ	−0.080**	−0.060*	−0.104***
	(0.035)	(0.034)	(0.035)
ln(GDP)		0.490***	0.593***
		(0.067)	(0.070)
ln(TP)		0.499*	0.452*
		(0.174)	(0.185)
FDI		2.816***	2.519***
		(0.671)	(0.663)
FDL		−0.034	0.013
		(0.026)	(0.026)
FE		0.310	−0.050
		(0.710)	(0.722)
GC			0.148
			(0.280)
EPI			0.118
			(0.183)
City FE	YES	YES	YES
Year FE	YES	YES	YES
_cons	6.339***	1.045	−1.023
	(0.008)	(1.329)	(1.372)
N	2538	2500	2363
r2_a	0.895	0.899	0.903

Note: Numbers in parentheses represent robust standard errors. *p < 0.1, **p < 0.05, ***p < 0.01. The empirical results are clustered at the city level. The same below.

### 5.2 Pre-trend test results

**Fig. 3:**
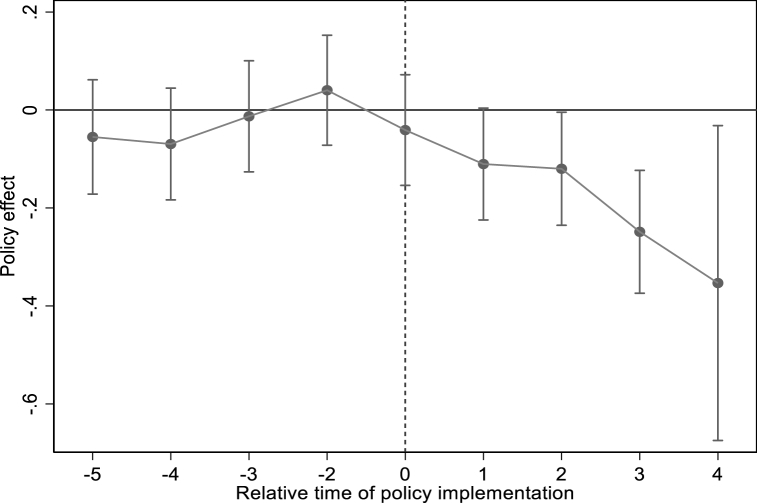
Pre-trend test of carbon emissions.

Fig. 3 shows the results of the pre-trend test.^3^ The estimated values of coefficients before establishing the NBDCPZ are not significant which has passed the pre-trend test. After implementing the NBDCPZ pilot policy, the estimated coefficients of NBDCPZ are significantly consistently negative. That is to say, establishing the NBDCPZ has sustainability in reducing CE.

### 5.3 Spillover effect test results

Table 3 reports the total and decomposed results based on model (6) under the geographical weight matrix ($W_{1}$) and economic weight matrix ($W_{2}$).^4^ The estimation coefficients (ρ) are not significant under the geographical weight matrix ($W_{1}$) nor under economic weight matrix ($W_{2}$), which preliminarily indicates that establishing the NBDCPZ has no spillover impact on the surrounding areas. However, the estimation coefficients (ρ) do not accurately reflect the spatial impact of NBDCPZ pilot policy. Therefore, we further calculated decomposed results of this pilot policy. Table 3 shows that under $W_{1}$ and $W_{2}$, the direct effects of implementing the pilot policy are significantly negative, while the indirect effects are not significant. This results further show that establishing the NBDCPZ only has an effect in reducing local CE only, thus the establishment of model (1) is reasonable.

**Table 3:** Spillover effect test results.

	W	NBDCPZ
ρ	$W_{1}$	0.449 (.262)
	$W_{2}$	−0.015 (0.076)
Direct effect	$W_{1}$	−0.185*** (0.054)
	$W_{2}$	−0.105** (0.054)
Indirect effect	$W_{1}$	0.590 (0.400)
	$W_{2}$	−0.038 (0.083)
Total effect	$W_{1}$	0.405 (0.368)
	$W_{2}$	−0.142** (0.064)

### 5.4 Robustness tests

#### 5.4.1 Staggered DID analysis of heterogeneity treatment effect

Under the staggered DID method, the estimated value of the double interaction term is the weighted average of the average treatment effect of different treatment groups in different periods [67]. However, because the weight of some treatment groups may be negative, the estimation results based on TWFE form can cause estimation errors [68]. For this reason, we use the DID robust estimators proposed by Callaway and Sant'Anna (2021) to solve the estimation error problem caused by the heterogeneity processing effect under the staggered DID method [69]. Columns (1)–(3) in Table 4 show the doubly robust DID estimators based on three different methods, respectively. After considering the heterogeneous processing effect, it can be found that the estimated coefficients of NBDCPZ are all significantly negative, which further confirms the reliability of the conclusions.

**Table 4:** Heterogeneity Treatment and PSM-DID estimation results.

VARIABLES	(1)	(2)	(3)	(4)	(5)	(6)
	LN(TCE)	LN(TCE)	LN(TCE)	LN(TCE)	LN(TCE)	LN(TCE)
NBDCPZ	−0.167**(0.067)	−0.139**(0.067)	−0.140**(0.069)	−0.101***(0.038)	−0.084**(0.035)	−0.097***(0.036)
Control variable	YES	YES	YES	YES	YES	YES
City FE	YES	YES	YES	YES	YES	YES
Year FE	YES	YES	YES	YES	YES	YES
**N**	2363	2328	2363	1623	2349	2039

#### 5.4.2 PSM-DID estimation

The selection process may be affected by unobserved factors, which may increase the “noise” of policy evaluation. To solve the influence of sample selection bias, we use the PSM-DID method to retest the benchmark regression results. First, we selected economic development level (ln(GDP)), total population (ln(TP)), openness (FDI), financial development level (FDL) and government intervention level (FE) as matching variables, using the nearest neighbor matching, nuclear matching and radius matching method to match year by year. Then, the DID is estimated using PSM matching samples. Columns (4)–(6) in Table 4 show that establishing the NBDCPZ has significantly reduced the CE.

#### 5.4.3 Placebo test

**Fig. 4:**
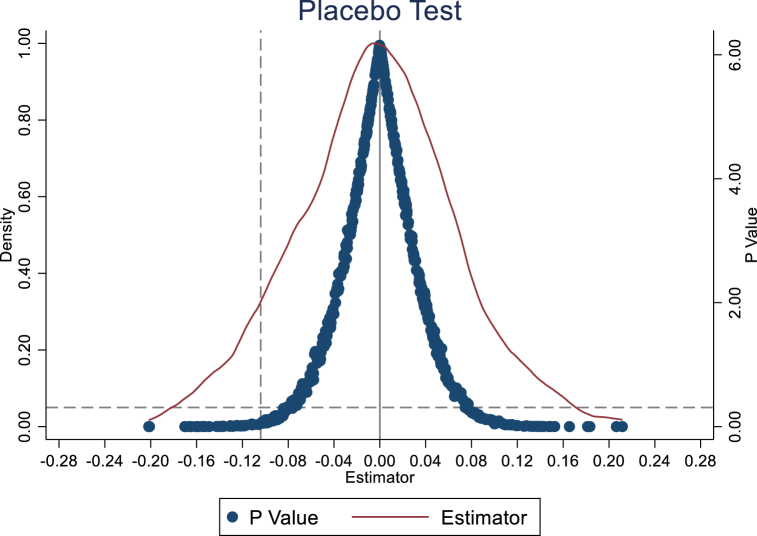
Placebo test of carbon emissions.

To further exclude accidental impacts, we also constructed a placebo test. We randomly set the implementation time of NBDCPZ in each region and randomly select NBDCPZ. Theoretically, the " pseudo " NBDCPZ pilot policy should not significantly impact carbon emissions. To this end, we repeat the above random process 500 times to estimate the model, and draws the nuclear density distribution and *P* value of the estimated coefficients of “pseudo” NBDCPZ (As shown in Fig. 4). The mean values of the estimated coefficients under the two random processes are near 0 and obey normal distribution. Most of the estimated coefficients of “pseudo” NBDCPZ are insignificant. We should note that the estimated NBDCPZ using ordinary sample locates on the edge of the simulated coefficients’ density plot, as indicated by dashed vertical line in Fig. 4. Combining these facts, we conclude that the effect of NBDCPZ to CE is not accidental, and our research conclusions are reliable and robust.

#### 5.4.4 Other robustness tests

In addition, we also conducted four robustness tests. The results are shown in Table 5 respectively. First, exclude other policy shocks. Considering the sample range of this study, many policies and shocks may impact CE. By sorting out the critical reform measures and relevant research in the same period, this paper believes that the Broadband China strategy (BCS) and Low-carbon City Pilot policies (LAC) will most likely affect the empirical results. To this end, we also control the corresponding policy dummy variables. Second, considering that China has not officially released unified carbon dioxide emissions data, we use the carbon emissions data published in CEADs as the explained variables to exclude the interference of different measurement methods of the explained variables on the regression results. Third, delete the central city. The behavior patterns of local city governments may differ, and the resources controlled by cities at higher administrative levels are far more than those of ordinary cities. Therefore, using all city samples to regress together may lead to certain deviations. For this reason, this paper will only retain the samples of ordinary prefecture-level cities for regression. Fourth, exclude the impact of extreme values. Considering the possible extreme values, all variables in the study sample were shrunk to 1% and 5%, respectively. The model (1) was regressed again. The above results confirm that the research conclusion has mostly stayed the same.

**Table 5:** Other robustness test results.

VARIABLES	(1)	(2)	(3)	(4)	
	LN(TCE)	LN(TCE)	LN(TCE)	LN(TCE)	LN(TCE)
NBDCPZ	−0.112 ***(0.035)	−0.038*(0.021)	−0.068*(0.036)	−0.093***(0.034)	−0.074**(0.031)
BCS	−0.062 *(0.015)				
LAC	−0.101 **(0.026)				
Control variable	YES	YES	YES	YES	YES
City FE	YES	YES	YES	YES	YES
Year FE	YES	YES	YES	YES	YES
**N**	2363	1797	2328	2363	2363
r2_a	0.904	0.959	0.895	0.905	0.910

## 6 Mechanism verification

### 6.1 Mediating effect of IST

**Fig. 5:**
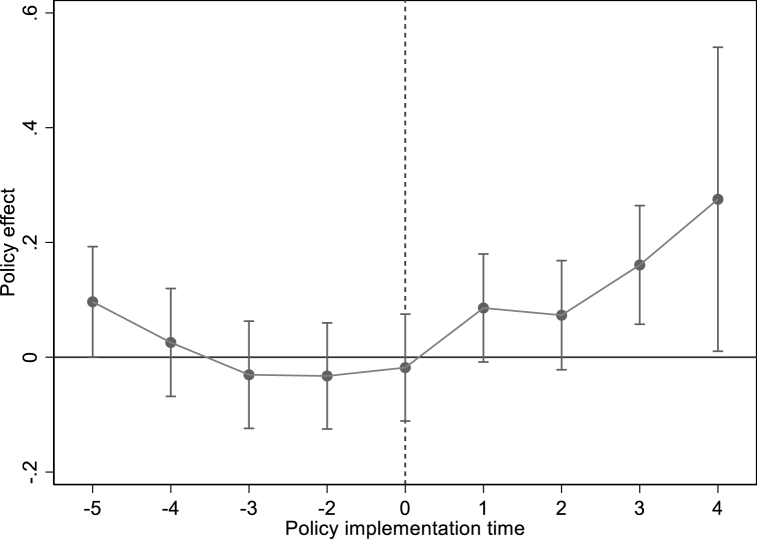
Pre-trend test of industrial structure upgrading.

The effect of NDT on CE through the intermediary role of ISU and ISO is shown in^5^ Table 6. In column (1), the regression coefficient of NBDCPZ is significantly negative, which means that NBDCPZ pilot policy significantly reduces the total CE. In column (2), the NBDCPZ pilot policy has significantly promoted ISU. In column (3), the impact of ISU on total CE is significantly negative. In column (4), we can see that the coefficient of NBDCPZ decreases after adding the ISU variable, which proves ISU is an important impact mechanism. At the same time, we also use model (5) to study the dynamic effect of NBDCPZ pilot on ISU .^6^ Fig. 5 shows that before the city becomes the NBDCPZ pilot city, the coefficients of NBDCPZ are all insignificant, while after joining the NBDCPZ, the role of NBDCPZ pilot policy in promoting ISU has gradually improved. This result further verifies that NBDCPZ pilot policy has indeed promoted ISU. In columns (5) and (6), NBDCPZ and ISO are not significant, which indicates that establishing the NBDCPZ does not reduced the total CE by promoting ISO. To summarize, NDT can reduce total CE by promoting IST. However, the intermediary role of this IST is mainly reflected in promoting ISU during the current sample period, which also^7^ verifies hypothesis 2.

**Table 6:** The mediating effect of IST.

VARIABLES	(3)	(3)	(3)	(4)	(5)	(6)
	LN(TCE)	ISU	LN(TCE)	LN(TCE)	ISO	LN(TCE)
NBDCPZ	−0.104*** (0.035)	0.060** (0.029)		−0.100*** (0.035)	−0.246 (0.417)	
ISU			−0.060** (0.027)	−0.057** (0.027)		
ISO						−0.001 (0.002)
Control variable	YES	YES	YES	YES	YES	YES
City FE	YES	YES	YES	YES	YES	YES
Year FE	YES	YES	YES	YES	YES	YES
**N**	2363	2363	2363	2363	2363	2363
r2_a	0.903	0.860	0.902	0.903	0.030	0.902

### 6.2 Mediating effect of GTI

**Fig. 6:**
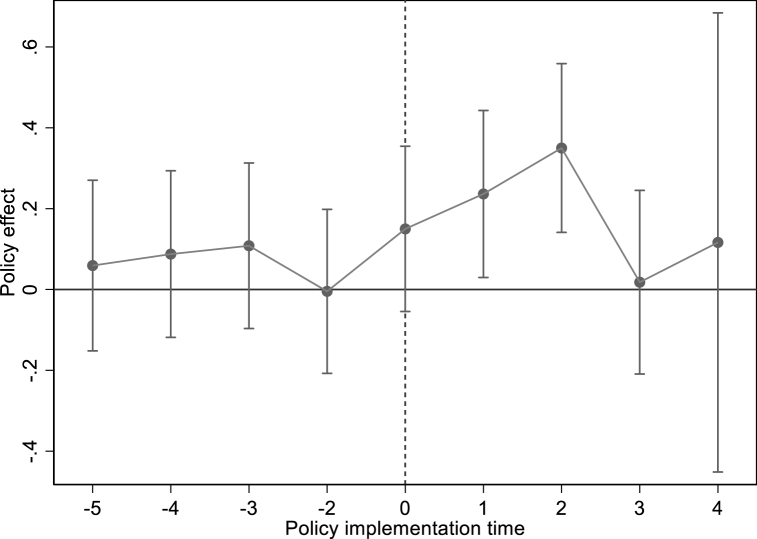
Pre-trend test of green technology innovation.

Table 7 shows the results of the impact of NDT on CE through the intermediary role of GTI. In column (1), NBDCPZ is significantly negative, meaning that NBDCPZ pilot policy significantly reduces the total CE of pilot cities. In column (2), the NBDCPZ pilot policy has significantly promoted GTI. In column (3), GTI is significantly negative, meaning that GTI can significantly reduce total CE. In column (4), we can see that the coefficient value of NBDCPZ decreases after adding the GTI variable, which proves GTI is an important impact mechanism. In addition, we also use model (5) to study the dynamic effect of NBDCPZ pilot on GTI. Fig. 6 shows that before the city becomes the NBDCPZ pilot city, the coefficients of NBDCPZ are all insignificant. In contrast, after implementing the pilot policy, the promotion of NBDCPZ pilot policy on GTI is enhanced first and then fades away. This result shows that the effect of NBDCPZ on GTI during the sample period is mainly reflected in the first few years of policy implementation. The reason for this phenomenon may be that the impact of NDT on GTI also has a marginal efficiency diminishing effect. That is to say, over time, the difficulty of GTI will become higher, and the impact of NDT will weaken. To sum up, NDT can reduce CE by promoting GTI, which also verifies hypothesis 3.

**Table 7:** The mediating effect of GTI.

VARIABLES	(1)	(2)	(3)	(4)
	LN(TCE)	GTI	LN(TCE)	LN(TCE)
NBDCPZ	−0.068** (0.035)	0.151** (0.063)		−0.055* (0.035)
GTI			−0.089*** (0.012)	−0.088** (0.012)
Control variable	YES	YES	YES	YES
City FE	YES	YES	YES	YES
Year FE	YES	YES	YES	YES
**N**	2327	2327	2327	2327
r2_a	0.894	0.800	0.897	0.897

### 6.3 Mediating effect of IA

**Fig. 7:**
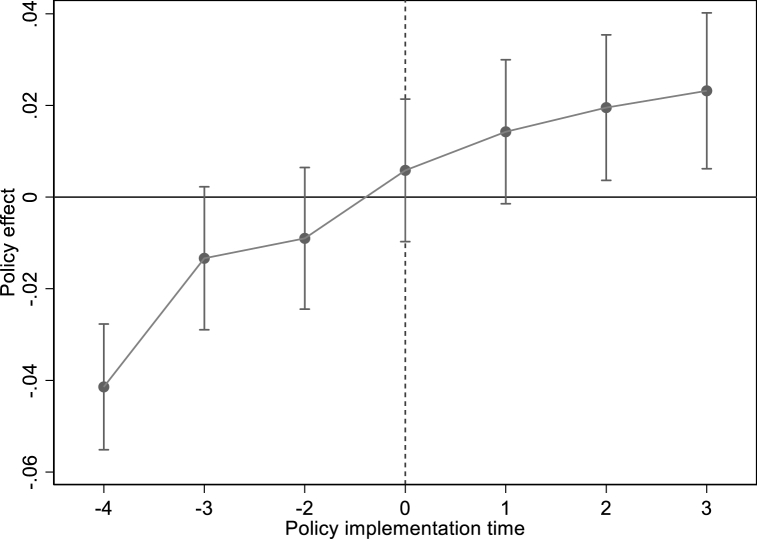
Pre-trend test of industrial agglomeration.

Table 8 shows the results of the impact of NDT on CE through the intermediary role of IA. In column (1), NBDCPZ is significantly negative, meaning NBDCPZ pilot policy significantly reduces total CE of pilot cities. In column (2), the NBDCPZ pilot policy can significantly improve the degree of IA. In column (3), IA is significantly negative, meaning IA can significantly reduce total CE. In column (4), The coefficient value of NBDCPZ decreases after adding the IA variable, which proves IA is an important impact mechanism. At the same time, we also use model (5) to study the dynamic effect of NBDCPZ pilot on IA. Fig. 7 shows that before the city becomes the NBDCPZ pilot city, the coefficients of NBDCPZ are almost all insignificant. After implementing the pilot policy, the role of NBDCPZ pilot policy in promoting IA has gradually increased. This result shows that NBDCPZ can improve the IA in the sample period. In conclusion, NDT can reduce CE by improving IA, which also verifies hypothesis 4.

**Table 8:** The mediating effect of IA.

VARIABLES	(1)	(2)	(3)	(4)
	LN(TCE)	IA	LN(TCE)	LN(TCE)
NBDCPZ	−0.104*** (0.035)	0.035*** (0.004)		−0.085** (0.035)
IA			−0.601*** (0.185)	−0.516*** (0.012)
Control variable	YES	YES	YES	YES
City FE	YES	YES	YES	YES
Year FE	YES	YES	YES	YES
**N**	2363	2363	2363	2363
r2_a	0.903	0.994	0.903	0.903

## 7 Heterogeneity analysis

### 7.1 Differences in urban location

Due to the differences in geographical location, climate characteristics, energy conservation, and environmental protection awareness, location differences may lead to different reactions to new digital technologies in different cities. Given this, we further test the heterogeneous effect of NDT on CE. Specifically, we divide the sample into the western and non-western regions and the southern and the northern regions, to test the location differential impact of NDT on CE. In columns (1) and (2) of Table 9, the NBDCPZ pilot policy significantly reduces the CE in the non-western regions. However, it cannot significantly affect the CE in the western regions. The possible reason is that the non-western regions have apparent advantages in capital, technology, talents, education, and other aspects, which provides a virtual environment and conditions for the NDT to quickly and effectively play the IUT effect, GTI effect, and IA effect. In columns (3) and (4), the NBDCPZ pilot policy significantly reduces the total CE in the southern regions. However, it cannot significantly affect the CE in northern regions. The possible reason is that the regional technology market in the southern regions is more complete, and the market environment is more open, which is very conducive to the IUT effect, GTI effect, and IA effect of NDT.

**Table 9:** Regression analysis results of differences in urban location.

VARIABLES	Western	Non-Western	Southern	Northern
	(1)	(2)	(3)	(4)
	LN(TCE)	LN(TCE)	LN(TCE)	LN(TCE)
NBDCPZ	−0.035 (0.091)	−0.107*** (0.037)	−0.187*** (0.051)	−0.063 (0.051)
Control variable	YES	YES	YES	YES
City FE	YES	YES	YES	YES
Year FE	YES	YES	YES	YES
**N**	660	1703	1305	1058
r2_a	0.881	0.908	0.913	0.891

### 7.2 Differences in urban size

The size of the city itself may also lead to differences in the carbon reduction effects of NDT. On the one hand, compared with small cities, large cities have significant advantages in terms of industrial structure, government capital investment, and transportation level, which can effectively allocate resources by using the economic agglomeration effect to solve the problem of CE better. However, larger cities also have strong demand for energy consumption. They need to consume many land resources, resulting in congestion effects and aggravating urban CE. Then, do NDT promote the economic agglomeration effect of cities to be greater than the congestion effect to reduce CE? Based on the population and economic size, we divide the sample into three sub-samples to test the city-size heterogeneity of the impact of NDT on CE.^8^ Table 10 shows that the NBDCPZ pilot policy has significantly reduced CE of cities with a larger population and economic scale. In contrast, the impact on LN(TCE) of small-scale cities is insignificant, indicating that the emission reduction effect of new digital technology in the current sample period is more significant in large-scale cities. The possible reason is that large-scale cities have suitable governance modes and huge talent advantages, which can further play the role of NDT in the industrial upgrading effect, green technology innovation effect, and industrial agglomeration effect.

**Table 10:** Regression analysis results of differences in urban size.

VARIABLES	size of population			size of economy		
	(1)	(2)	(3)	(4)	(5)	(6)
	S	M	L	S	M	L
	LN(TCE)	LN(TCE)	LN(TCE)	LN(TCE)	LN(TCE)	LN(TCE)
NBDCPZ	0.008 (0.077)	−0.017 (0.065)	−0.224*** (0.048)	0.100 (0.100)	0.025 (0.063)	−0.108** (0.050)
Control variable	YES	YES	YES	YES	YES	YES
City FE	YES	YES	YES	YES	YES	YES
Year FE	YES	YES	YES	YES	YES	YES
**N**	765	800	796	748	781	794
r2_a	0.874	0.876	0.936	0.815	0.843	0.885

## 8 Research conclusions and policy recommendations

Based on the panel data of 282 prefecture-level cities in China from 2011 to 2019, this paper uses the quasi-natural experiment of the NBDCPZ pilot policy to empirically examine the environmental effects of NDT, analyze their mechanisms affecting CE, explain the heterogeneity of the above effects, and provide empirical evidence for accelerating the development of NDT globally, especially in developing countries. The research results show that: first, NDT significantly reduce CE. This conclusion is still valid after a series of robustness tests such as heterogeneity treatment effect analysis, ex-ante trend test, spillover effect test, and placebo test. Second, the mechanism verification results show that the new digital technologies can reduce carbon emissions by promoting the IST, improving the level of GTI, and promoting IA. Third, the heterogeneity analysis shows that new digital technologies' CE reduction effect is more evident in non-western regions, southern regions, and large cities.

Through this study, we can draw three policy recommendations. First, China should further promote the rapid development of new digital technologies based on the development practice of the NBDCPZ. The role of digital technologies in economic development has been widely recognized, and the conclusions of this paper further demonstrate that new digital technologies with data elements at the core can contribute to reducing carbon emissions. Therefore, new digital technologies should be taken as an essential starting point to achieve the goals of " dual carbon” and high-quality economic development, and the development and application of new digital technologies should be comprehensively promoted. Second, China should implement policies to support and promote industrial structure transformation, enterprise R&D and innovation, and industrial agglomeration. Industrial structure transformation, green technology innovation, and industrial agglomeration are the main channels for new digital technologies to reduce carbon emissions. Therefore, when guiding the development and application of new digital technologies, the government should provide more specific supportive services for relevant enterprises to promote the agglomeration of enterprises, enhance their green technology innovation ability and promote the industrial structure optimization and upgrading. Third, governments should adapt to local conditions, consider the different characteristics of urban development, and give full play to new digital technologies' economic and environmental benefits by implementing dynamic and differentiated policies. The heterogeneity analysis shows that the development and application of new digital technologies have different environmental welfare effects in different geographical locations and city sizes. Therefore, the government should make targeted policies and plans according to local geographical characteristics, population size, and economic scale, and avoid blindly copying the development experience of other regions, thereby making the new digital technologies with data elements as the core to maximize the environmental welfare effect.

## Author contribution statement

Han BU conceived and designed the experiments, performed the experiments, analyzed and interpreted the data, contributed reagents, materials, analysis tools or data, and wrote the paper; Guomin LI analyzed and interpreted the data, and contributed reagents, materials, analysis tools or data; Xiangyu YU contributed reagents, materials, analysis tools or data; Zhou XUN conceived and designed the experiments, performed the experiments, analyzed and interpreted the data, contributed reagents, materials, analysis tools or data, and wrote the paper.

## Data availability statement

Data will be made available on request.

## Additional information

Supplementary content related to this article has been published online at [URL].

## Declaration of competing interest

The authors declare that they have no known competing financial interests or personal relationships that could have appeared to influence the work reported in this paper.

## Figures and Tables

**Table A1:** Results of regression model in the second stage

VARIABLES	(1)	(2)	(3)	(4)	(5)
	LN(TCE)	LN(TCE)	LN(TCE)	LN(TCE)	LN(TCE)
$\hat{ISU}$	−1.736*** (0.584)			−2.082** (1.047)	0.883 (0.974)
$\hat{GTI}$		−0.457** (0.237)		0.238 (0.218)	0.126 (0.169)
$\hat{IA}$			−2.947*** (0.991)		−5.800*** (3.439)
Control variable	YES	YES	YES	YES	YES
City FE	YES	YES	YES	YES	YES
Year FE	YES	YES	YES	YES	YES
**N**	2363	2327	2363	2327	2327
r2_a	0.903	0.895	0.903	0.895	0.895

## Appendix A

Parallel trend refers to the fact that the experimental group has the same time variation trend as the control group without receiving treatment, which is the prerequisite for correctly identifying causal effects by the DID model. Since the counterfactual cases cannot be observed, the parallel trend hypothesis cannot be directly tested. Therefore, we investigate whether the prior trends in the observable trial and control groups are the same. Given this hypothesis, we use the event study method Jacobson et al. (1993) proposed for the pre-trend test [70]. The specific model setting is as follows^9^:

(5) $$\mathrm{LN}\;{\left(TCE\right)}_{ct}=\partial_{0}+\sum_{k=-4,k\neq -1}^{k=3}\partial_{k}{NBDCPZ}_{ck}+\gamma z_{ct}+\tau_{t}+\delta_{c}+\varepsilon_{ct}$$

where k represents the window period of the event study method. ${NBDCPZ}_{ck}$ is a set of dummy variables. If city c is set up as the NBDCPZ in the k phase, the value is 1; otherwise, it is 0. The value of $\partial_{k}$ reflects the difference in the CE in pilot and non-pilot cities of the NBDCPZ before and after the implementation of the policy.

## Appendix B

Another necessary condition for the DID model is to satisfy the stable unit treatment values assumption (SUTVA), that is, whether different individuals are affected by policies independently of each other. If the NBDCPZ affects CE in all cities, the DID model cannot accurately assess the causal effect. Given this hypothesis, we use the spatial DID model proposed by Dub é et al. (2014) and the practice of Jia et al. (2021) to construct a spatial Dubin model-based DID model for the spillover effect test [71,72]. The specific model setting is as follows:

(6) $$\mathrm{LN}\;{\left(TCE\right)}_{ct}=\partial_{0}+\theta \sum_{j}w_{cj}\;\mathrm{LN}\;{\left(TCE\right)}_{ct}+\partial_{1}{NBDCPZ}_{ct}+\rho \sum_{j}w_{cj}{NBDCPZ}_{ct}+\gamma z_{ct}+\varphi \sum_{j}w_{cj}z_{ct}+\tau_{t}+\delta_{c}+\varepsilon_{ct}$$

where $w_{cj}$ is an element of the spatial weight matrix W, which describes the spatial proximity between cities, the value of ρ reflects whether the NBDCPZ pilot policy has a spillover effect.

## Appendix C

The intermediary effect model brings endogenous problems due to introducing intermediary variables. To further strengthen the impact mechanism, we use a two-stage regression model, which can alleviate the endogenous problems introduced by intermediary variables to a certain extent. The specific model setting is as follows:

(7) $${MID}_{ctk}=\delta_{0}+\delta_{1}{NBDCPZ}_{ct}+\vartheta z_{ct}+\tau_{t}^{1}+\delta_{c}^{1}+\psi_{ct}$$

(8) $$\mathrm{LN}\;{\left(TCE\right)}_{ct}=\beta_{0}+\beta_{1}\hat{{MID}_{ctk}}+\omega z_{ct}+\tau_{t}^{2}+\delta_{c}^{2}+\varsigma_{ct}$$

Where, $\hat{{MID}_{ctk}}$ is the predicted values for model (8) and other variable settings are consistent with model (5) and model (6).

Table (A1) shows the results of the second stage of the two-stage regression model. Column (1) shows that $\hat{ISU}$ is significantly negative, further strengthening the IST effect of NDT in CE reduction. Column (2) shows that $\hat{GTI}$ is significantly negative, which further verifies the GTI effect of NDT in CE reduction^10^. At the same time, column (3) shows that $\hat{IA}$ is significantly negative, which further strengthens the IA effect of NDT in CE reduction.

## Footnotes

1 The White Paper on the Development of China's Digital Economy (2021).

2 For the implementation background of this policy, see 2.2.

3 The method of pre-trend test is shown in Appendix A.

4 The method of Spillover effect test is shown in Appendix B.

5 The specific method is shown in model (2–4). The subsequent sections on mechanism analysis have all utilized this method.

6 In all second step of Mediating effect analysis, as in model (5), we apply the same DID strategy to ensure the correct causality.

7 At the same time, we also utilized a two-stage model to further validate the mechanism, and the analysis results are presented in Appendix C.

8 We divide the sample into three sub-samples based on 1/3 quantile and 2/3 quantile: small cities (S), medium cities (M) and large cities (L).
